# Associations of Plasma p‐tau181 With Age, Adjusted for Kidney Function and Sociodemographic Factors

**DOI:** 10.1002/gps.70138

**Published:** 2025-07-29

**Authors:** Jemma Hazan, Kathy Y. Liu, Henrik Zetterberg, Nick Fox, Robert Howard, with ADNI

**Affiliations:** ^1^ Division of Psychiatry University College London London UK; ^2^ Dementia Research Institute University College London London UK; ^3^ Institute of Neurology University College London London UK; ^4^ Department of Psychiatry and Neurochemistry Institute of Neuroscience and Physiology the Sahlgrenska Academy at the University of Gothenburg Mölndal Sweden; ^5^ Clinical Neurochemistry Laboratory Sahlgrenska University Hospital Mölndal Sweden; ^6^ Hong Kong Center for Neurodegenerative Diseases Hong Kong China; ^7^ Wisconsin Alzheimer's Disease Research Center University of Wisconsin School of Medicine and Public Health University of Wisconsin‐Madison Madison Wisconsin USA

**Keywords:** Alzheimer's disease, dementia, diagnosis, p‐tau, plasma biomarkers

## Abstract

**Introduction:**

Plasma phosphorylated tau (p‐tau) levels, such as p‐tau181, are elevated in Alzheimer's disease compared to cognitively unimpaired individuals. They represent potential candidate blood biomarkers for use in memory services where CSF examinations are not available. However, the effect of age on plasma p‐tau levels remains undetermined. Limited studies have investigated the association between age and plasma p‐tau thus far, and fewer still have differentiated levels by brain amyloid pathology. Characterising these associations and determining if this is influenced by sociodemographic factors or medical comorbidities is important for establishing blood biomarker reference ranges.

**Methods:**

Using ADNI data, we analysed 860 observations (581 participants; age range: 55–95 years; 56.0% male; 93.6% White). Linear mixed models (LMMs) estimated fixed effects of age, creatinine, baseline BMI, sex, ethnicity, and group (Control vs. AD) on plasma p‐tau181 concentration, with a random intercept for participant ID. Separate LMMs assessed covariate effects and interactions with group status.

**Results:**

Analysis of ADNI data revealed a significant positive association between p‐tau181 levels, group status, and creatinine in the fully adjusted LLM. Group status may have obscured the total effect of age on p‐tau181, as its removal from the model resulted in a significant age effect. Single‐variable models showed the positive association between either age, or creatinine and p‐tau181 levels did not differ between control and AD groups. There was a significant negative association between BMI and plasma p‐tau, which was stronger in AD versus control groups.

**Conclusions:**

This study provides insights into the factors that may influence plasma p‐tau181 levels. These findings underscore the need to account for clinical and demographic factors when interpreting p‐tau181. Future research should validate these associations in diverse populations and explore underlying mechanisms.

## Key Points

- ADNI data revealed a significant positive association between p‐tau181 levels, group status, and creatinine in the fully adjusted LLM. Group status may have obscured the total effect of age on p‐tau181, as its removal from the model resulted in a significant age effect. Single‐variable models showed the positive association between either age, or creatinine and p‐tau181 levels did not differ between control and AD groups. There was a significant negative association between BMI and plasma p‐tau, which was stronger in AD versus control groups.
- A comprehensive approach is needed to interpret plasma p‐tau181 levels in diverse, older populations with comorbidities, considering clinical context and validating influencing factors.

**Abbreviations**
AD: Alzheimer’s disease
ADNI: Alzheimer’s Disease Neuroimaging Initiative
A+: Amyloid‐PET positive
A‐: Amyloid‐PET negative
CU: Cognitively unimpaired
CI non‐AD: Cognitively impaired non‐AD
95%CI: 95% Confidence Intervals
IQR: Interquartile range
MCI: Mild cognitive impairment
PSEN1: Presenilin 1 gene
NIA‐AA: National Institute on Ageing and Alzheimer’s Association
SD: standard deviation
SUVR: Standardised uptake value
WHICAP: Washington Heights‐Inwood Columbia Ageing Project
↑: Increased
+: Positive
‐: negative

## 1 Introduction

Plasma phosphorylated (p‐tau) proteins are among the leading blood biomarkers for the detection of AD pathology [1]. The development of ultrasensitive immunoassays provides a means of measuring plasma p‐tau at ultra‐low concentrations in the blood [2, 3]. Plasma p‐tau is strongly associated with both extracellular amyloid plaques and neurofibrillary tau tangle brain pathology, reflected by amyloid‐positron emission tomography (amyloid‐PET), tau‐positron emission tomography (tau‐PET), cerebrospinal fluid (CSF) measures and post‐mortem examinations [3, 4, 5, 6, 7]. Plasma p‐tau shows good discrimination between AD, cognitively unimpaired individuals, and those with other neurodegenerative disorders [1, 7, 8, 9].

Following recent marketing approvals in the U.S. for amyloid‐lowering therapies, the Alzheimer's Association have proposed updated criteria for the biological definition of Alzheimer's disease [10]. This has led to a need to confirm the presence of AD brain pathology by in vivo biomarker measures [11]. Most dementia diagnoses occur in patients assessed at community memory services, which serve a diverse and older population [12]. AD Blood‐based biomarkers represent a more scalable alternative to methods such as CSF analysis or amyloid‐PET, which require specialist equipment and training [12]. Their use in community clinics is especially promising given the improved performance of newer plasma biomarker assays [13]. Confirming AD biological status will be essential for both participant trial inclusion and selection of AD patients to receive disease‐modifying treatments [14].

A PubMed literature search using the terms (plasma AND p‐tau AND tau) identified studies exploring associations between p‐tau and age, with inclusion criteria of peer‐reviewed, published studies in English. Included studies reported on plasma p‐tau217 or plasma p‐tau181 levels using an ultra‐sensitive immunoassay, as well as age. Seven cohort studies reported on the association of plasma p‐tau levels with age, with characteristics and main findings are summarised in Table S1. These studies identified age, renal function, body mass index (BMI), ethnicity and cardiovascular risk as factors potentially influencing p‐tau181 levels [7, 15, 16, 17, 18, 19, 20]. It is important to note that short‐term inter‐individual variability in plasma p‐tau181 biomarker assays, potentially due to analytical imprecision or biological fluctuations, may affect the reliability of diagnostic interpretation in AD and Control groups [21].

However, the association between p‐tau181 and age is inconsistent, particularly in amyloid‐negative individuals, and its interaction with amyloid status remains underexplored. Bouteloup et al. found age as a primary driver of AD biomarker variance in cognitively unimpaired cohorts [22]. Factors such as ethnicity, kidney function, cardiovascular risk, sex, and BMI may also influence p‐tau181 levels. Clarifying these associations is vital for establishing diagnostic test accuracy and reference ranges in community settings, where patients often exceed 80 years and have comorbidities.

This study investigates the influence of age, renal function, BMI, ethnicity, sex and amyloid pathology on plasma p‐tau181 levels using the Alzheimer's Disease Neuroimaging Initiative (ADNI) dataset, informed by the literature search, to contribute to blood biomarker reference ranges for AD diagnosis in community settings.

## 2 Methods

### 2.1 Study Design and Data

We used anonymised data available from ADNI (ADNI‐LONI [23]) database (adni.loni.usc.edu). The ADNI was launched in 2003 as a public‐private partnership, led by Principal Investigator Michael W. Weiner, MD. The primary goal of ADNI has been to test whether serial magnetic resonance imaging (MRI), positron emission tomography (PET), other biological markers, and clinical and neuropsychological assessment can be combined to measure the progression of mild cognitive impairment (MCI) and early AD. The ADNI study is approved by regional ethical committees. For further information, see www.adni‐info.org.

**FIGURE 1:**
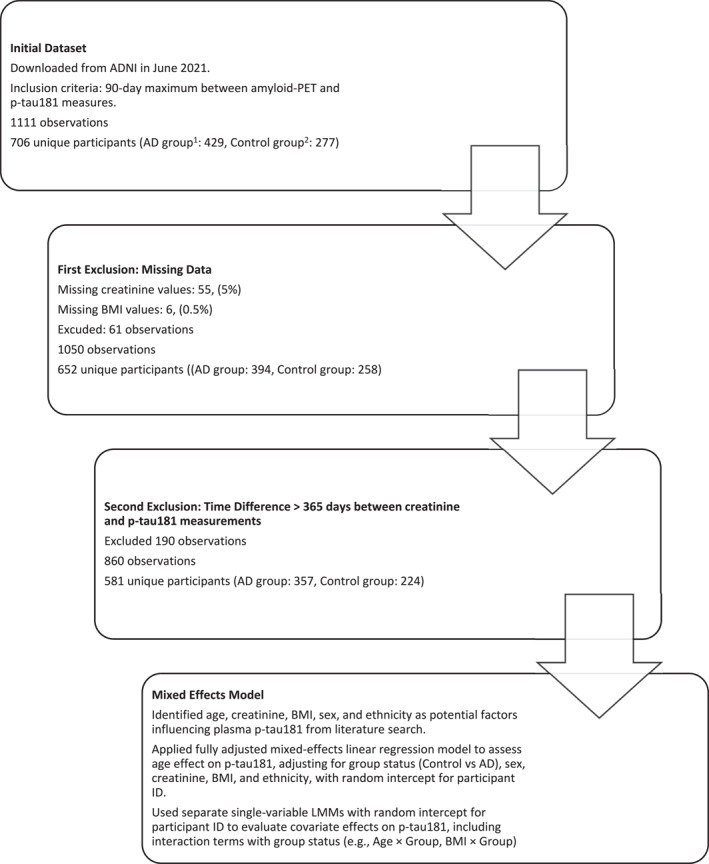
Study flow diagram of data inclusion and statistical analyses. 1 AD Group: Amyloid‐PET 18F‐florbetapir Standardised Uptake Value Ratio (SUVR) ≥ 1.11 and Clinical Dementia Rating Sum of Boxes (CDR‐SB) global scores > 0. 2 Control group: Amyloid‐PET 18F‐florbetapir SUVR < 1.11 and CDR‐SB = 0.

We analysed a subset of an ADNI cohort, comprising 1111 observations from 706 participants in a previous study [17]. This was filtered to 860 observations (581 unique participants; AD: 357, Control: 224) after excluding missing data values for creatinine (55, 5%) and BMI (6, 0.5%), and > 365 days between creatinine and p‐tau181 measurements (18.1%) and as detailed in the study flow diagram (Figure 1).

Participants were stratified by amyloid‐PET (18F‐florbetapir), Standardised Uptake Value Ratio (SUVR) ≥ 1.11 and Clinical Dementia Rating Sum of Boxes (CDR‐SB) global scores [24] > 0 for AD group and SUVR < 1.11, CDR‐SB = 0 for control group with a 90‐day maximum between amyloid‐PET and p‐tau181 (Single Molecule array, University of Gothenburg [3]). The data was longitudinal with repeated measures available for some participants who had several available time‐matched measures that fitted within the defined parameters (90‐day maximum duration between paired amyloid‐PET and plasma p‐tau181 assessments).

Predictors, identified from the literature search, included age, creatinine Nightingale Health's NMR metabolomics platform [25] (each p‐tau181 participant visit was time‐matched with the closest available creatinine level by date), BMI, sex, ethnicity, disease, and stroke.

### 2.2 Statistical Analyses

BMI was recorded at the baseline visit, and ethnicity was comprised of two categories: White or ‘minority ethnic group’, which included American Indian or Alaskan Native, Asian, Black or African American, Native Hawaiian or Other Pacific Islander and More Than One Race groups. We chose to combine these minority ethnic groups as individual groups were relatively small (range *n* = 1–29). We had also aimed to investigate the cardiovascular risk on p‐tau levels, however the data subset did not include participants with a history of stroke.

Missingness tests, specifically Little's MCAR test, indicated that data were Missing Completely at Random (MCAR) for p‐tau181 (*p* = 0.340) and suggested weak Missing at Random (MAR) for sex and age (*p* = 0.06 each), supporting the use of listwise deletion with minimal bias. Sensitivity analyses evaluated the robustness of key findings across a larger dataset (*n* = 1050) before excluding > 365 days between creatinine and p‐tau181 measurements, an imputed dataset (*n* = 1111) using k‐nearest neighbours (*k* = 5) to assess the impact of missing data.

Model assumptions (linearity, homoscedasticity, no multicollinearity [variance inflation factors (VIFs) < 1.3]) were assessed using residual plots for linearity and homoscedasticity, VIFs for multicollinearity, and the Shapiro‐Wilk test (*W* = 0.828, *p* < 0.001) for residual normality. A (log (p‐tau181 + 1)) was employed in sensitivity analyses to address non‐normal p‐tau181 data (Shapiro‐Wilk *W* = 0.828, *p* < 0.001), showing consistent effect directions and significance, thereby confirming the robustness of the untransformed LMM results despite residual non‐normality. Additionally, a linear regression model (*n* = 860) was included in the sensitivity analyses to compare with the LMM, assessing the impact of the random intercept for participant ID on covariate effects. The linear regression, without the random intercept, had similar estimates to the LMM, though with slightly wider confidence intervals, indicating the LMM's appropriateness.

Age, creatinine, BMI, sex and ethnicity were identified as factors that could also potentially influence plasma p‐tau181 concentration from relevant studies during the literature search.

We first used a mixed‐effects multivariable linear regression model (the ‘fully adjusted model’) to estimate effects of age on plasma p‐tau181 concentration adjusting for group status (Controls vs. AD), sex, creatinine, BMI and ethnicity, with a random intercept for participant ID. Mixed‐effects models can account for both fixed effects and random effects associated with intra‐individual variation with repeated measures.

Next we used separate single‐variable LMMs with a random intercept for participant ID to evaluate the fixed effects of age, creatinine, BMI, sex, and ethnicity on plasma p‐tau181 levels in the ADNI cohort, incorporating interaction terms with Group status (Control vs. AD) to assess whether these effects vary by group (e.g., Age × Group (Control vs. AD), BMI × Group (Control vs. AD)).

All analyses were performed using *R* Software *R* version 4.1.2; *R* Foundation for Statistical Computing [26].

## 3 Results

### 3.1 Demographics

The study sample included 581 unique participants, with 357 AD (amyloid‐PET SUVR ≥ 1.11, CDR‐SB > 0) and 224 Control (SUVR < 1.11, CDR‐SB = 0) participants. Demographic characteristics (Table 1) showed a median age of 74 years (IQR: 69–79), 55.9% male, 93.1% White, median BMI of 26.6 kg/m^2^ (IQR: 24.3–29.5) and a median creatinine of 73.6 μmol/L (IQR: 64.4–84.5).

**TABLE 1:** A summary of study characteristics and main findings.

Characteristic	Controls (*n* = 224)	AD (*n* = 357)	All (*n* = 581)
	Median (IQR) or *n* (%)	Median (IQR) or *n* (%)	Median (IQR) or *n* (%)
Age (years)	73 (68–78)	75 (70–80)	74 (69–79)
Sex (male)	115 (51.3%)	210 (58.8%)	325 (55.9%)
Race			
White	205 (91.5%)	336 (94.1%)	541 (93.1%)
BMI (kg m^2^)	27.4 (24.7, 30.8)	26.1 (23.9, 29.0)	26.6 (24.3, 29.5)
Creatinine (μmol/L)	72.8 (63.9, 83.5)	74.4 (65.0, 85.0)	73.6 (64.4, 84.5)

### 3.2 Fully Adjusted Multivariable Model

This model was employed to assess the impact of age on plasma p‐tau181 levels while adjusting for multiple covariates, including creatinine, BMI, sex, group status, and ethnicity, with a random intercept for participant ID to account for within‐subject correlations. The model revealed a non‐significant effect of age on p‐tau181 (*β* = 0.13, 95% CI: −0.00 to 0.25, *p* = 0.05). Significant effects were observed for creatinine (*β* = 0.06, 95% CI: 0.01 to 0.12, *p* = 0.03), indicating a positive association with p‐tau181, and BMI (*β* = −0.29, 95% CI: −0.48 to −0.11, *p* = 0.00), reflecting a negative association. Group status showed a highly significant effect, with AD group participants exhibiting elevated p‐tau181 levels compared to controls (*β* = 8.01, 95% CI: 6.14 to 9.87, *p* < 0.001). Neither sex (*p* = 0.68) nor ethnicity (*p* = 0.22) demonstrated significant associations with p‐tau181 levels in this adjusted model. To address potential circularity between disease status (defined by amyloid‐PET and cognition) and p‐tau181, a sensitivity analysis excluding disease status was conducted. This model revealed a significant effect of Age (*β* = 0.15, 95% CI: 0.02 to 0.29, *p* = 0.03). Further information is available in Table 2.

**TABLE 2:** Mixed effects regression analyses examining the association between p‐tau181 levels and group, age, creatinine, BMI, sex, and minority ethnic group.

Model Covariate(s)	Regression coefficients (95% confidence intervals)	Interaction term coefficient: Group (Control vs. AD) and (95% confidence intervals)^d^
**Dependent Variable:** p‐tau181 (plasma p‐tau181 levels) **Independent variables:** Age, creatinine, BMI, sex, ethnicity (minority ethnic group), group (Control vs. AD) (fixed effects); participant ID (random effect to address correlations) **Model:** Linear mixed model (LMM) with random intercept for participant ID to account for within‐subject correlations		
Group (Control vs. AD)		
Age	0.22 (−0.09, 0.34) *	0.08 (−0.18, 0.35)
Creatinine^a^	0.13 (0.05, 0.22) **	−0.09 (−0.20, 0.02)
BMI	−0.05 (−0.36, 0.26) *	−0.39 (−0.77, −0.00) *
Sex (female vs. male)^b^	−1.85 (−4.80, 1.09)	0.83 (−2.97, 4.63)
Ethnicity (minority ethnic group)^c^	−5.45 (−10.77, −0.13)	4.56 (−2.74, 11.86)
Age + creatinine + BMI + sex + ethnicity + group		
Age	0.13 (−0.00, 0.25)	
Creatinine	0.06 (0.01, 0.12) *	
BMI	−0.29 (−0.48, −0.11) **	
Sex (female vs. male)	−0.40513 (−2.35, 1.54)	
Ethnicity (minority ethnic group)	−2.29 (−5.92, 1.33)	
Group (AD vs. Control)	8.01 (6.14, 9.87) ***	

* *p*‐value map 0 ‘***’ 0.001 ‘**’ 0.01 ‘*’ 0.05.

^a^ Serum Creatinine (μmol/L).

^b^ Male reference value.

^c^ White reference value.

^d^ Interaction terms (e.g., Age: Group) represent the additional effect of the covariate across Group levels (Control vs. AD), calculated as the product of the variables.

### 3.3 Single Variable Interaction Models

To explore group‐specific effects, separate LMMs were fitted for each covariate with an interaction term for group status (Control vs. AD).

### 3.4 Age

The interaction between age and disease status was not significant (*p* = 0.53).

### 3.5 Renal Function

For the ADNI data, the mean duration between creatinine and p‐tau blood draw dates was 29.6 (SD 44.9) days. There was no significant interaction between creatinine and group status (*p* = 0.09).

### 3.6 BMI

The interaction between BMI and group status was significant (−0.39 pg/mL per kg/m^2^, 95% CI: −0.77, −0.00, *p* = 0.049), indicating a weaker negative effect in controls compared to AD participants.

### 3.7 Ethnicity

The interaction with group status was not significant (*p* = 0.22).

### 3.8 Sex

Sex did not demonstrate a significant interaction with group status (*p* = 0.67).

## 4 Discussion

### 4.1 Summary of Findings

This study used linear mixed models (LMMs) within the ADNI cohort to examine the effects of age on p‐tau181 while adjusting for creatinine, BMI, sex, ethnicity, and group status (Control vs. AD) as identified from a literature search. We also assessed whether there was a group effect on any association between each individual variable and p‐tau181.

In the fully adjusted LLM, AD group status and creatinine showed a significant positive association with plasma p‐tau181 levels, and BMI showed a significant negative association with p‐tau181. Group status may have obscured the total effect of age on p‐tau181, as its removal from the model resulted in a significant age effect. Single‐variable models showed that the positive association between either age or creatinine and plasma p‐tau did not differ between control and AD groups. The negative association between BMI and plasma p‐tau was stronger in AD versus control groups.

### 4.2 Comparison With Previous Literature

Our findings align with prior studies on plasma p‐tau181 associations. Seven studies reported on p‐tau181 and age, with five finding a positive association in cognitively impaired participants [7, 15, 17, 18, 27] and one study reported no association [20] between p‐tau and age. For cognitively unimpaired participants, five studies reported a positive association [16, 17, 18, 20, 27] and one study reported no association [7] between p‐tau and age.

In terms of methods used, four studies performed a regression analysis of age on plasma p‐tau levels [16, 18, 20, 27], one study performed a Pearson's rank association test [15], and one study used log‐transformed plasma p‐tau217 levels fitted to a restricted cubic spline mode [7].

Only two studies examined age‐related differences in plasma p‐tau by amyloid‐PET status [17, 18], and findings in these studies were inconsistent. Mielke [18] et al.’s findings support the concept that age‐related increases in plasma p‐tau181 and p‐tau217 are relatively specific to amyloid positivity. They noted that the primary increase with age was among individuals with elevated brain amyloid and that there were small increases with age among those without elevated brain amyloid. In contrast, Hazan [17] et al., who used plasma p‐tau181 measures from the ADNI cohort, did not perform a regression analysis, instead presenting a plotted chart that revealed an overlap of 95% confidence intervals for AD and control participants over 85 years.

There were key differences between these studies in terms of participant characteristics and methods (Table S2). Mielke et al. categorised CU, MCI and dementia participants by clinical diagnosis and a subset by amyloid‐PET positivity, whereas Hazan et al. categorised controls, cognitively impaired AD or non‐AD participants based on amyloid‐PET positivity and CDR‐SB score. The distribution of characteristics within diagnostic categories therefore differs, with Mielke et al. including a larger proportion of participants without cognitive symptoms. The studies used different amyloid positivity cutoffs: Mielke et al. defined amyloid positivity with an Aβ Pittsburgh compound B PiB‐PET tracer and a SUVR of 1.48, while Hazan et al. used a 18F‐florbetapir Aβ‐PET (AV45) tracer with a SUVR of 1.11. Although the age effect and the other factors examined were statistically significant, the change in p‐tau concentration associated with amyloid positivity in cognitively impaired individuals was far more pronounced. Therefore, compared to amyloid positivity or disease status, these factors they are likely to have a relatively minor clinical impact.

Further work in other independent datasets is needed to establish if there is a clinically important association, and if specific age‐related reference ranges are required. Age‐specific reference ranges have been produced for another blood‐based biomarker, serum neurofilament light, where levels have been positively associated with age [28].

The molecular pathways associated with plasma p‐tau and increasing age have not been fully explained. Age‐related amyloid‐β accumulation is associated with increased plasma p‐tau, particularly p‐tau217 and p‐tau231 [29]. Ageing may also promote tau hyperphosphorylation through oxidative stress and impaired protein degradation [30]. Blood‐brain barrier changes could increase tau release into plasma [31], while reduced clearance may contribute to p‐tau accumulation in both the brain and plasma [32].

The significant creatinine p‐tau181 association in our study is consistent with reports of higher p‐tau181 in chronic kidney disease (CKD). Impaired renal function is believed to affect plasma p‐tau levels via reduced clearance of p‐tau from the blood, resulting in elevated levels [33]. Mielke et al. reported that chronic kidney disease (CKD) and cardiovascular disease were associated with higher plasma p‐tau measures, with the CKD effect greater than the age effect [18]. Pan et al. report that in cognitively normal participants, those with chronic kidney disease were more likely to have increased plasma p‐tau181 levels [20].

BMI may also affect plasma p‐tau levels [34]. The negative association between p‐tau181 and higher BMI is thought to reflect a dilutional effect.

This negative BMI association aligns with Brickman et al. who reported that increased BMI was associated with lower plasma p‐tau181 and p‐tau217 levels [15]. However, Mielke et al. reported that the association between BMI and p‐tau levels was not significant after age adjustment and sex adjustment [18].

Minority ethnic group did not show a significant association with plasma p‐tau181 levels. Brickman et al. selected a cohort with equal proportion of ethnic groups, which included non‐Hispanic White (White), Hispanic, and non‐Hispanic Black/African American participants. They reported similar p‐tau levels amongst the three ethnic groups [18].

Mielke et al. reported that there were no significant differences in plasma p‐tau181 levels by sex in the entire cohort [18]. We also found that sex did not demonstrate a significant association with ptau181 in the ADNI cohort.

Several medical comorbidities may influence plasma p‐tau levels as they are risk factors for AD or affect physiological processes [18, 35]. Mielke et al. used a real‐world community population and included participants with a range of medical comorbidities: depression, hypertension, diabetes, stroke, myocardial infarction (MI), atrial fibrillation, head trauma and cancer [18]. They reported that stroke and MI were associated with increased plasma p‐tau levels in age‐ and sex‐adjusted participants. As our ADNI sample did not include participants with a history of stroke, analysis of cardiovascular health status was not conducted.

These findings highlight the interplay of renal, metabolic, and demographic factors in p‐tau181 variability, necessitating tailored clinical guidelines for AD biomarker use in diverse, ageing populations.

### 4.3 Limitations

The literature search was limited by requiring both ‘plasma’ and ‘p‐tau’ as search terms and focussing on studies reporting associations with age. The combined ‘plasma p‐tau’ term may have excluded studies using alternative terms, while the association focus, chosen for comparability with our linear mixed‐effects model analyses, may have omitted null findings or non‐associational designs, introducing publication bias and restricting contextual evidence. Using serum creatinine instead of eGFR as a kidney function marker avoids multicollinearity with age and sex but has limitations. eGFR, preferred clinically for standardised adjustments [36], may be more robust, while creatinine varies with muscle mass or diet [37].

Limitations in the ADNI data analyses were that the participants' BMI values were only included at baseline visit, with an average of 603 days between the baseline BMI visit and p‐tau measure. The time difference between creatinine and p‐tau181 measurements was constrained to ≤ 365 days (mean = 29.6, SD = 44.9 days), and observations with missing serum creatinine (55, ∼5%) or BMI (6, ∼0.5%) values were omitted to ensure complete and temporally relevant data for linear mixed modelling. However, even this shorter creatinine–p‐tau gap may affect associations due to potential renal function changes with the passage of time, given the possible renal clearance of p‐tau181 [38]. Although, plasma p‐tau levels are relatively stable [18], and significant renal decline typically occurs over the course of years rather than months. Missing data values for serum creatinine and BMI had minimal bias, as indicated by missingness tests suggesting missing completely at random (MCAR) or weak missing at random (MAR) mechanisms. We were unable to examine cardiovascular risk factors in the ADNI analysis, which might have partly accounted for the effect of age or creatinine on plasma p‐tau.

Further research is needed to study the age‐adjusted effect of co‐morbidities such as high BMI, poor renal function, cardiac conditions and prior stroke, on plasma p‐tau levels. No studies recorded the disease duration of participants. This may have an important influence of plasma p‐tau levels. A limitation of this study was the reliance on a single database, albeit one that provides data from a global, large, and diverse cohort. Prospective longitudinal cohort studies using real‐world populations with data on disease duration, co‐morbidities, sociodemographic factors, and amyloid status may more robustly address knowledge gaps on the association between p‐tau and age.

## 5 Conclusion

This study provides insights into the factors that may influence plasma p‐tau181 levels. The results may help refine reference ranges and interpretation guidelines, though further validation is needed. A considered approach is required to effectively prepare clinicians to interpret plasma p‐tau in a clinical setting, where most patients seen are in the older age category, are ethnically diverse and have multiple medical co‐morbidities. This is to ensure that the blood biomarker level is interpreted holistically in the context of the clinical picture. There are currently limitations in their application with further prospective validation work to confirm the association of co‐variates on plasma p‐tau levels. The use of educational tools to accompany biomarker results and provide information on the context and limitations of p‐tau values will be essential.

## Author Contributions

All authors contributed to the conception and design of the review. JH wrote the first draft and all authors revised and approved the article for publication.

## Conflicts of Interest

HZ has served at scientific advisory boards and/or as a consultant for Abbvie, Acumen, Alector, Alzinova, ALZPath, Amylyx, Annexon, Apellis, Artery Therapeutics, AZTherapies, Cognito Therapeutics, CogRx, Denali, Eisai, Merry Life, Nervgen, Novo Nordisk, Optoceutics, Passage Bio, Pinteon Therapeutics, Prothena, Red Abbey Labs, reMYND, Roche, Samumed, Siemens Healthineers, Triplet Therapeutics, and Wave, has given lectures in symposia sponsored by Alzecure, Biogen, Cellectricon, Fujirebio, Lilly, Novo Nordisk, and Roche, and is a co‐founder of Brain Biomarker Solutions in Gothenburg AB (BBS), which is a part of the GU Ventures Incubator Programme (outside submitted work). NF has served at scientific advisory boards and/or as a consultant for Biogen, Eisai, Ionis, Lilly, Roche/Genentech, and Siemens.

## Supporting information

Table S1

Table S2

## Data Availability

The data that support the findings of this study are available from the corresponding author upon reasonable request.
