# Supplementary material for: Associations of Plasma p‐tau181 With Age, Adjusted for Kidney Function and Sociodemographic Factors
Source: Int J Geriatr Psychiatry. 2025 Jul 29;40(8):e70138. doi: 10.1002/gps.70138 (PMC12306926; doi:10.1002/gps.70138)
Supplement: Supplementary file 1 — Table S1 [file GPS-40-e70138-s002.docx]

| **Study Author** | **Year of Publication** | **Study Design** | **Publication Type** | **Cohort Region** | **Cohort** | **Plasma**  **p-tau measure** | **Sample size** | **Age**  **(yrs)** | **Participant groups** | **Participant status definition** | **Data Analysis** | **Study findings** |
| --- | --- | --- | --- | --- | --- | --- | --- | --- | --- | --- | --- | --- |
| Brickman et al | 2020 | Cohort | Paper | USA | WHICAP | P-tau181, P-tau217 | 300 | Mean (+/-SD) 85.64 (+/-7.13) | Clinically evaluated | Committee defined: AD, control, other dementia. | Pearson correlations and t-tests. | ↑ age associated with ↑ ptau217 (r = 0.192, P = .001) & ptau181 (r = 0.214, P < .001). |
| Chen et al | 2023 | Cohort | Paper | China | Community elderly population | P-tau 181 | 193 | Mean (+/-SD) 67.3 (+/-9.8) | CU | status defined by MMSE score ≥27. | Linear regression | P-tau181 levels ↑ with age, r=0.305, P< 0.001 |
| Hazan et al | 2021 | Cohort | Paper | USA | ADNI | P-tau 181 | 964 | Mean (+/-SD) 74.9 (+/-7.2) | 429 AD, 256 CI non-AD & 279 controls. | A+ cutoff ≥1.11 SUVR. AD= CDR-global >0 & A+. CI non-AD = CDR >0 and A-. Control= CDR-global=0 and A- | P-tau-181 data & 95%CI. Significance testing non-paired, 2-tailed student-t tests. | P-tau181 levels differentiated participants, robust up to 85yrs. For 85-95yrs 95%CIs overlapped |
| Mielke et al | 2022 | Cohort | Paper | USA | Mayo clinic Study of Ageing Cohort | P-tau181,  P-tau217 | 1329 | Median (IQR): 73.2 (53.5, 81.3) | Clinical diagnosis: 1161 CU, 153 MCI, 15 dementia | Amyloid-PET + cutoff ≥1.48 SUVR | Regression analysis | P-tau181 & p-tau217 levels ↑ with age. P-tau ↑ for A+ vs A− participants ( P-tau181: 0.43 versus 0.06, P< 0.001 & 7.2-fold ↑. P-tau217: (0.48 versus 0.04, P < 0.001) & 12 fold greater ↑ |
| Ossenkoppele et al | 2021 | Cohort | Paper | Sweden | Biofinder 2 | P-tau181, P-tau217 | 400 | Mean (+/-SD) 67.7 (+/-11.4) | 219 CU, 181 MCI or AD (NIA-AA criteria). | Cognitive status definition: CU & symptomatic groups (MCI & dementia). | Ridge regression model | Plasma p-tau181 and p-tau217 levels were independent of tau PET associated with higher age |
| Palmqvist et al | 2020 | Cohort | Paper | Colombia | The Alzheimer Prevention Initiative Colombia Registry | P-tau217 | 662 | Mean (+/-SD) 35.8 (+/-10.7) | 365 (59%) PSEN1 mutation carriers. 257 (41%) noncarriers | CI participants, PSEN1 carriers & matched from the same kindred. | P-tau217 levels fitted to restricted cubic spline model separately for PSEN1 mutation carriers/ noncarriers. | Mutation carriers p-tau217 levels ↑ with age. Non carriers p-tau217 levels did not increase with age. |
| Pan et al | 2023 | Cohort | Paper | China | Shanghai hospital population | P-tau 181 | 685 | Median age (IQR) 65 (60, 70) | 401 (CU)  284 (CI). | CI participants = standardized neuropsychological test performance > 1 SD below the age-corrected normative mean. | Linear regression analysis | CU: p-tau181 positively correlated with age and Aβ-PET SUVR. CI: p-tau181 not positively correlated with Aβ-PET SUVR |

**Supplementary Table.1. Participant Characteristics Table**
