# Supplementary material for: Associations of Plasma p‐tau181 With Age, Adjusted for Kidney Function and Sociodemographic Factors
Source: Int J Geriatr Psychiatry. 2025 Jul 29;40(8):e70138. doi: 10.1002/gps.70138 (PMC12306926; doi:10.1002/gps.70138)
Supplement: Supplementary file 2 — Table S2 [file GPS-40-e70138-s001.docx]

**Supplementary Table 2: A summary of Hazan et al.**^18^ **and Mielke et al.**^23^ **study characteristics**

| Study characteristic(s) | Hazan et al. | Mielke et al. |
| --- | --- | --- |
| Participants (n) | 923 | 1329 |
| Age (yrs) range | 55.0-95.0 | 30.7- 97.9 |
| Age (yrs) median (interquartile range) | 75 (70.0-80.0) | 73.2 (53.5, 81.3) |
| Plasma p-tau measure | p-tau181 (Simoa, University of Gothenburg assay) | p-tau217, p-tau181  (MSD platform by electrochemiluminescence, Lilly Research Laboratories) |
| Participant definition | ¹Categorical criteria | ²Clinical criteria |
| Participant categories | 1) Controls (n=277)  2) Cognitively impaired AD (n=429)  3) Cognitively impaired non-AD (n=217). | 1) CU (n=1161)  2) MCI (n=153)  3) Dementia n=15  (10 with AD dementia, 2 with Lewy body dementia, 1 with other dementia and 2 indeterminate). |
| Participants with amyloid-PET (n) | 923 | 1051 |
| Amyloid-PET tracer | 18F-florbetapir Aβ-PET (AV45) | Aβ Pittsburgh compound B (PiB)-PET |
| Amyloid-PET SUVR positivity cut off | ≥1.11 | ≥1.48 |
| Amyloid positive cases | Cognitively impaired AD (n=429) | 1) CU: 303/892 (34.0%)  2) MCI: 99/144 (68.8%)  3) Dementia: 12/15 (80.0%). |

Abbreviations: AD, Alzheimer’s disease; amyloid-PET, amyloid positron emission tomography; CDR-SB score, CDR Sum of Boxes; CU, cognitively unimpaired; MCI, mild cognitive impairment; MSD, Meso Scale Discovery; p-tau, plasma phosphorylated tau; Simoa, Single Molecule Array; SUVR, standardized uptake value ratio

¹Control= amyloid-PET negative & CDR-SB score = 0, Cognitively impaired AD= amyloid-PET positive and a CDR-SB score of >0

²CU, MCI, Dementia
